# Correlation Between Patient Satisfaction and Surgeon Evaluation of Long-Term Nasolabial Outcomes in Unilateral Cleft Lip Repair

**DOI:** 10.7759/cureus.88169

**Published:** 2025-07-17

**Authors:** Muath Mamdouh Mahmod Al-Chalabi, Wan Azman Wan Sulaiman, Ahmad Sukari Halim

**Affiliations:** 1 Reconstructive Sciences Unit, Universiti Sains Malaysia (USM), Kota Bharu, MYS

**Keywords:** cleft lip repair, nasolabial appearance, patient’s satisfaction, unilateral cleft lip, unilateral cleft lip and palate

## Abstract

Background: Achieving a well-balanced and symmetrical facial appearance is a primary objective in cleft lip and palate repair. The long-term nasolabial appearance serves as a key indicator of surgical success. This study aims to assess the correlation between patient satisfaction and surgeon evaluation of nasolabial outcomes following unilateral cleft lip repair. It explores whether patient satisfaction may serve as a reliable indicator of long-term surgical success.

Methods: A retrospective cross-sectional study was conducted using records of patients with unilateral cleft lip who underwent surgical repair by the Reconstructive Science Unit at Universiti Sains Malaysia Hospital. Inclusion criteria required that patients had undergone lip repair before the age of two and were at least 14 years old at the time of follow-up assessment. A total of 50 patients met these criteria and were included in the analysis. Surgeon assessments and patient-reported satisfaction regarding nasolabial appearance were extracted and analyzed to evaluate their correlation.

Results: No significant differences were observed between surgeon assessments and patient-reported satisfaction with nasolabial appearance. A statistically significant association was found between surgeon evaluations and patient satisfaction (χ²(1) = 14.881, p < .001), indicating that higher surgeon-rated outcomes were associated with greater patient satisfaction.

Conclusion: This study demonstrates a significant correlation between surgeon evaluations and patient satisfaction with long-term nasolabial appearance, suggesting that patient satisfaction may serve as a practical and meaningful indicator for assessing long-term surgical outcomes in unilateral cleft lip repair.

## Introduction

Cleft lip and palate (CLP) are among the most prevalent congenital craniofacial anomalies worldwide, affecting approximately 1 in 700 live births [1]. Management aims include restoring facial symmetry and proportion, improving feeding, speech, and hearing, and enhancing facial appearance to minimize psychological distress for patients and families [2].

While CLP presents functional challenges, the long-term cosmetic outcome, particularly the nasolabial appearance, remains a major concern. The long-term nasolabial appearance becomes especially relevant as final facial features emerge after skeletal maturity. Residual facial asymmetries may lead to long-term social and psychological consequences, including negative self-perception and reduced facial attractiveness [3,4]. Traditional assessments of cleft surgery outcomes have focused on objective metrics such as anatomical measurements, photographic evaluations, morbidity, and mortality rates [5]. Despite surgical repair, many patients present with residual deformities involving the lip, nose, and dentition. Additionally, the intervention itself may contribute to secondary growth disturbances, including nasal asymmetry, upper lip distortion, and scarring or flattening of the philtral groove [4].

Assessing treatment success in cleft management involves multiple domains, including facial aesthetics, dentofacial development, speech, nasal function, hearing, patient satisfaction, and overall quality of life. However, there remains no consensus among cleft care specialists regarding which outcome domains should be prioritized [6].

Perceptions of facial attractiveness are inherently subjective and vary widely among individuals. Over the past two decades, increasing attention has been given to the influence of ethnic differences in the perception and evaluation of beauty, emphasizing the need for surgeons to recognize and preserve facial features unique to specific ethnic groups [7]. Among adult patients, the appearance of the nose and upper lip, particularly nasal asymmetry, remains a primary aesthetic concern [8-10]. Individuals with visible facial differences are at risk for social exclusion, low self-esteem, communication difficulties, and distorted self-image [11]. Despite advances in surgical technique, achieving complete facial symmetry in cleft lip (CL) repair remains challenging [8]. Evaluating nasolabial appearance offers a valuable approach to assessing long-term aesthetic outcomes and guiding surgical refinements, potentially improving patient care, outcome prediction, and satisfaction [12].

Notably, satisfaction with long-term surgical outcomes differs from satisfaction with individual clinical encounters. Postoperative satisfaction is strongly influenced by patient-reported outcomes and the alignment between expectations and results [13].

This study aims to investigate long-term patient satisfaction with nasolabial appearance following unilateral cleft lip repair and to evaluate its correlation with surgeon assessments. We hypothesize that patient satisfaction may serve as a reliable indicator of long-term surgical success, thereby promoting patient-centered outcome evaluation in cleft care.

## Materials and methods

This retrospective study was conducted by the Reconstructive Science Unit at Universiti Sains Malaysia Hospital with institutional review board approval. Patients included had unilateral cleft lip (CL), underwent surgical repair using the Millard rotation-advancement technique within the first two years of life, and were at least 14 years old at the time of the follow-up assessment, as this age marks the completion of facial skeletal growth and a stable nasolabial appearance, enabling a more accurate evaluation of patient satisfaction. Patients were included regardless of whether the cleft was isolated or associated with a cleft alveolus or palate. Only those who had a single primary lip repair without any subsequent surgical interventions, such as revision surgery or rhinoplasty, were selected to ensure that outcomes reflected the initial procedure alone. Medical records from patients treated between 1997 and 2008 were manually retrieved from hospital archives. Patients with incomplete documentation, syndromic presentation, or a history of lip repair performed at an external center prior to referral were excluded.

At each follow-up, the attending plastic surgeon assessed the patient’s nasolabial appearance through direct clinical observation and recorded their evaluation using a predefined categorical scale. Surgeon ratings were classified into one of four categories: unsatisfactory appearance of both lip and nose, satisfactory appearance of both lip and nose, satisfactory appearance of the lip only, or satisfactory appearance of the nose only.

During the same visit, patients were also asked to provide feedback regarding their satisfaction with their facial appearance. Their responses were recorded by the attending plastic surgeon using the same four predefined categories applied to the surgeon’s own assessment. These patient responses were based on direct verbal feedback and subsequently categorized for analysis.

Statistical analysis was performed using IBM SPSS Statistics for Windows, Version 24 (Released 2016; IBM Corp., Armonk, New York). The relationship between surgeon and patient satisfaction ratings was examined using the Chi-squared (χ²) test. A one-sample t-test was also conducted to compare mean satisfaction levels between surgeons and patients. A p-value of < 0.05 was considered statistically significant.

## Results

Out of all records reviewed, 50 cases met the inclusion criteria, and additional cases were excluded due to incomplete data or syndromic presentations. The final sample included 50 patients: 13 males (26%) and 37 females (74%). All variables and assessment categories are summarized in Table 1.

**Table 1:** Cross-tabulation of surgeon and patient ratings of nasolabial appearance by clinical and demographic variables. Ratings were categorized into four groups: Unsatisfactory (both lip and nose), Satisfactory Lip Only, Satisfactory Nose Only, and Satisfactory (both lip and nose). CLP: cleft lip and palate, CL: cleft lip, CLA: cleft lip alveolus.

Variable	Surgeon rating				Patient rating			
	Unsatisfactory	Lip only	Nose only	Satisfactory	Unsatisfactory	Lip only	Nose only	Satisfactory
Sex								
Male	2	3	1	7	2	2	1	8
Female	13	10	8	6	13	11	7	6
Age at operation (months)								
3	5	7	4	8	7	6	3	8
4	4	5	3	2	3	5	3	3
5	4	0	1	2	4	0	1	2
6	0	1	1	0	0	1	1	0
7	0	0	0	1	0	0	0	1
13	1	0	0	0	1	0	0	0
15	1	0	0	0	0	1	0	0
Cleft diagnosis								
Left incomplete CLP	1	1	1	2	0	2	1	2
Left incomplete CLA	0	1	0	0	0	1	0	0
Left incomplete CL	0	0	1	1	0	0	1	1
Left complete CLP	6	6	8	7	6	4	10	7
Right incomplete CLP	0	0	0	0	0	0	0	0
Right incomplete CLA	0	1	0	0	0	1	0	0
Right incomplete CL	1	1	1	1	1	1	1	1
Right complete CLP	2	5	0	3	2	5	0	3
Family history of cleft								
No	10	10	6	9	8	11	6	10
Yes	5	3	3	4	7	2	2	4
Presence of secondary deformity								
None	0	0	0	13	0	0	0	13
Lip	0	0	9	0	1	0	8	0
Nose	0	13	0	0	1	12	0	0
Both	15	0	0	0	13	1	0	1

Female patients received fewer satisfactory ratings (64.9%) from surgeons compared to males (86.6%), considering the lip, nose, or both. Across all age groups at the time of surgery, both satisfactory and unsatisfactory evaluations were observed from surgeons and patients. Notably, all patients who underwent surgery after 12 months of age received unsatisfactory evaluations from both the surgeon and the patient. Patient satisfaction was reported across all cleft types, except in cases of left and right incomplete cleft lip alveolus (CLA). Dissatisfaction, however, was noted only in left complete CLP, right incomplete CL, and right complete CLP.

Patients with a family history of cleft had lower proportions of satisfactory ratings from both surgeons (66.7% vs. 71.4%) and patients (53.3% vs. 77.1%) compared to those without such a history. Surgeon assessments generally corresponded with the presence of secondary deformities such as nasal asymmetry, vermilion notching, lip shortening, or irregularities of the philtrum or nasal base. Interestingly, a small number of patients expressed satisfaction with their appearance despite the presence of these secondary deformities.

To assess the relationship between surgeon evaluations and patient satisfaction, a chi-squared test was performed. The analysis revealed a significant association between surgeon evaluations and patient satisfaction (χ²(1) = 14.881, p < .001). Surgeon ratings of satisfactory nasolabial appearance were significantly associated with higher levels of patient satisfaction, as shown in Table 2. A one-sample t-test was conducted to assess whether differences between surgeon and patient evaluations deviated significantly from zero. The test showed no significant difference between surgeon and patient evaluations (t(49) = -0.28, p = .780).

**Table 2:** Association between surgeon and patient ratings of nasolabial appearance

Surgeon’s rating	Patient unsatisfied	Patient satisfied	χ^2^	p
Unsatisfactory	8	2	14.881	< 0.001
Satisfactory	7	33		

## Discussion

Comprehensive cleft care extends beyond surgical correction to prioritize both functional restoration and an aesthetically acceptable outcome, particularly from the patient’s perspective [14]. In recent decades, with the goal of improving health-related quality of life, the assessment of appearance has increasingly focused on the patient’s perspective of received health care using questionnaires, collectively referred to as patient-reported outcome measures. Although there are many methods to assess facial and nasolabial aesthetics, such as two-dimensional photographs, three-dimensional images, videographic assessment, and direct clinical assessment, there is no internationally established, standardized, and uniform rating method for evaluating facial or nasolabial aesthetics in patients with cleft after surgical repair [5]. Additionally, evaluating patient satisfaction can be a good indicator of the provided management [15], which indirectly may play a role in determining the nasolabial appearance post-surgical repair of CLP.

Mani et al. [8] used a Satisfaction with Appearance scale (SWA), developed by the Psychology Special Interest Group of the Craniofacial Society of Great Britain and Ireland, for self-assessment of nasolabial appearance. They concluded that self-assessment among patients with clefts does not correlate with professional reviews. Taiwo et al. [15] reported 71.4% surgical success and 94.8% patient satisfaction, while our findings showed 80% surgeon-rated success and 82% patient satisfaction.

In assessing patient satisfaction, we conducted a cross-tabulation of surgeon and patient comments on nasolabial appearance based on different variables. Interestingly, surgeon satisfaction ratings were lower in female patients (64.9%) compared to males (86.6%) in terms of nasolabial appearance, including the lip, nose, or both. This may suggest that surgeons demonstrated more rigorous decision-making when commenting on nasolabial outcomes in female patients. However, the overall percentage of surgical results deemed acceptable by surgeons was the same as that of satisfied patients (70%).

When the age variable was assessed in relation to patient satisfaction with nasolabial appearance, satisfactory and unsatisfactory ratings were observed across most age groups. However, among patients who underwent surgery after 12 months of age, all received unsatisfactory ratings from both the surgeon and the patient. This finding supports the perspective that encourages early-age operations and adheres to the standard protocol to ensure the first lip repair is completed before the age of six months [16,17].

While surgeon assessments are routinely recorded following lip repair, incorporating the patient’s perspective is essential for a holistic evaluation of outcomes. An outcome may be evaluated as excellent based on clinical measurements and objective scales, while at the same time, it may be deemed poor and unsatisfactory by the patient. Chen et al. [18] reported that a multicenter study of outcomes following orthodontic treatment for unilateral CLP in 17-year-old patients revealed that 20% of patients were dissatisfied with their facial profile, as were their parents. In contrast, Rivera et al. [19] reported in a prospective cohort satisfaction study that satisfaction with medical care did not correspond with the fulfillment of expectations; parents were likely to report satisfaction even when their expectations went unfulfilled, with no comments mentioned regarding patient satisfaction. In comparison, our study focuses solely on patient satisfaction.

Based on our findings, which showed a significant association between surgeon and patient satisfaction, obtaining patient feedback on their subjective perspective may serve as a practical approach to assess long-term outcomes, especially when regular follow-up is difficult. Utilizing secure hospital communication systems to collect this feedback could offer an effective alternative in such cases. While the study is limited by a relatively small sample size, the results emphasize the value of patient-reported satisfaction in evaluating cleft lip repair outcomes. Future research should aim to refine subjective assessment tools and develop efficient strategies for remote follow-up.

## Conclusions

This study demonstrated a significant association between surgeon evaluations and patient satisfaction regarding nasolabial appearance following CL repair. The close alignment in satisfaction ratings supports the clinical value of incorporating patient perspectives into long-term outcome assessments. While patient satisfaction may reflect surgical success, especially during extended follow-up, further studies using standardized tools are recommended to confirm its reliability as an independent indicator of surgical success.
